# Unveiling the dynamics of gut microbial interactions: a review of dietary impact and precision nutrition in gastrointestinal health

**DOI:** 10.3389/fnut.2024.1395664

**Published:** 2024-05-30

**Authors:** Zifang Shang, Liu Pai, Sandip Patil

**Affiliations:** ^1^Guangdong Engineering Technological Research Center of Clinical Molecular Diagnosis and Antibody Drugs, Meizhou People's Hospital (Huangtang Hospital), Meizhou Academy of Medical Sciences, Meizhou, China; ^2^Department of Haematology and Oncology, Shenzhen Children’s Hospital, Shenzhen, China

**Keywords:** microbiome, gastrointestinal health, precision nutrition, gut-brain axis, autoimmune diseases

## Abstract

The human microbiome, a dynamic ecosystem within the gastrointestinal tract, plays a pivotal role in shaping overall health. This review delves into six interconnected sections, unraveling the intricate relationship between diet, gut microbiota, and their profound impact on human health. The dance of nutrients in the gut orchestrates a complex symphony, influencing digestive processes and susceptibility to gastrointestinal disorders. Emphasizing the bidirectional communication between the gut and the brain, the Brain-Gut Axis section highlights the crucial role of dietary choices in physical, mental, and emotional well-being. Autoimmune diseases, particularly those manifesting in the gastrointestinal tract, reveal the delicate balance disrupted by gut microbiome imbalances. Strategies for reconciling gut microbes through diets, precision nutrition, and clinical indications showcase promising avenues for managing gastrointestinal distress and revolutionizing healthcare. From the Low-FODMAP diet to neuro-gut interventions, these strategies provide a holistic understanding of the gut’s dynamic world. Precision nutrition, as a groundbreaking discipline, holds transformative potential by tailoring dietary recommendations to individual gut microbiota compositions, reshaping the landscape of gastrointestinal health. Recent advancements in clinical indications, including exact probiotics, fecal microbiota transplantation, and neuro-gut interventions, signify a new era where the gut microbiome actively participates in therapeutic strategies. As the microbiome takes center stage in healthcare, a paradigm shift toward personalized and effective treatments for gastrointestinal disorders emerges, reflecting the symbiotic relationship between the human body and its microbial companions.

## Introduction

The human body often likened to a complex ecosystem, vividly exemplifies this analogy in the form of the gut microbiome (1). Within the intricate landscape of the human digestive system, a bustling community of microorganisms, encompassing bacteria, viruses, fungi, and more, collectively orchestrates a symphony that profoundly influences human health and well-being (2, 3). In the context of article, “symphony” likely refers to the intricate and coordinated interactions within the gastrointestinal tract involving diet, gut microbiota, and their impact on health. The gut microbiome, a dynamic and diverse population, engages in a multifaceted relationship with its human host, contributing to various physiological processes and serving as a pivotal player in maintaining equilibrium (4). This microbial ecosystem within the digestive tract is not a static entity but rather a living, evolving ecology shaped by an interplay of factors such as genetics, diet, environment, lifestyle, and even the mode of delivery at birth (5, 6). The makeup of this intricate microbiome is composed of thousands upon thousands of microbial species delicately existing in a precarious equilibrium. The gut, acting as the primary residence for this diverse microbial community, houses an array of microorganisms, predominantly bacteria, alongside viruses, archaea, and eukaryotic species (1). Far from a passive bystander, the gut microbiota actively participates in key physiological processes that impact human health. Its role extends to the intricate breakdown of complex carbohydrates, proteins, and fats that might challenge the body’s enzymes (7). The trillions of bacteria populating the digestive tract play a vital role in breaking down these molecules into absorbable forms, facilitating nutrient absorption (8). Moreover, the gut microbiota exerts influence over metabolic processes, affecting energy storage, nutrient processing, and appetite regulation. A symbiotic relationship is evident in the microbiota’s contribution to immune system modulation. By conditioning the immune system to respond effectively to harmful pathogens while curbing unnecessary inflammation, the gut microbiota acts as a crucial ally in maintaining immune balance (9). Additionally, certain microbial inhabitants are involved in the synthesis of essential vitamins B and K, as well as short-chain fatty acids (SCFAs) renowned for their anti-inflammatory properties (10, 11). Preserving the delicate equilibrium of the gut microbiome, termed dysbiosis- (disruption in the gut microbiota composition when disrupted), is paramount for overall health. Dysbiosis has been linked to various diseases and conditions, including irritable bowel syndrome (IBS), inflammatory bowel disease (IBD), obesity, and certain neurological disorders (12). Therefore, maintaining a diverse and stable microbial community is integral to promoting a healthy gut microbiome. Dietary choices emerge as a powerful tool in shaping the gut microbiome. Consuming meals rich in dietary fiber fosters an environment conducive to the thriving of beneficial bacteria. Prebiotic foods, encompassing fibers that serve as sustenance for beneficial bacteria, contribute to microbial diversity and further support gut health. In essence, the gut microbiome stands as a complex ecosystem with far-reaching effects on human health (13). From its pivotal role in digestion to its contribution to immune function, understanding the profound symbiotic link between humans and their microbial inhabitants underscores the significance of the gut microbiome. Elevating awareness of its importance and making informed dietary choices to promote diversity within this microbial community hold the potential to enhance health outcomes and deepen our comprehension of this intricate relationship. This burgeoning field of microbiome research is poised to transform our approach to human health, paving the way for innovative therapeutic interventions and personalized treatments targeting the gut microbiome. As we delve deeper into the intricacies of microbiome dynamics in human diseases, the potential for groundbreaking discoveries and therapeutic breakthroughs becomes increasingly apparent.

## Navigating the nutrient landscape: impact on gut microbiota

**Figure 1:**
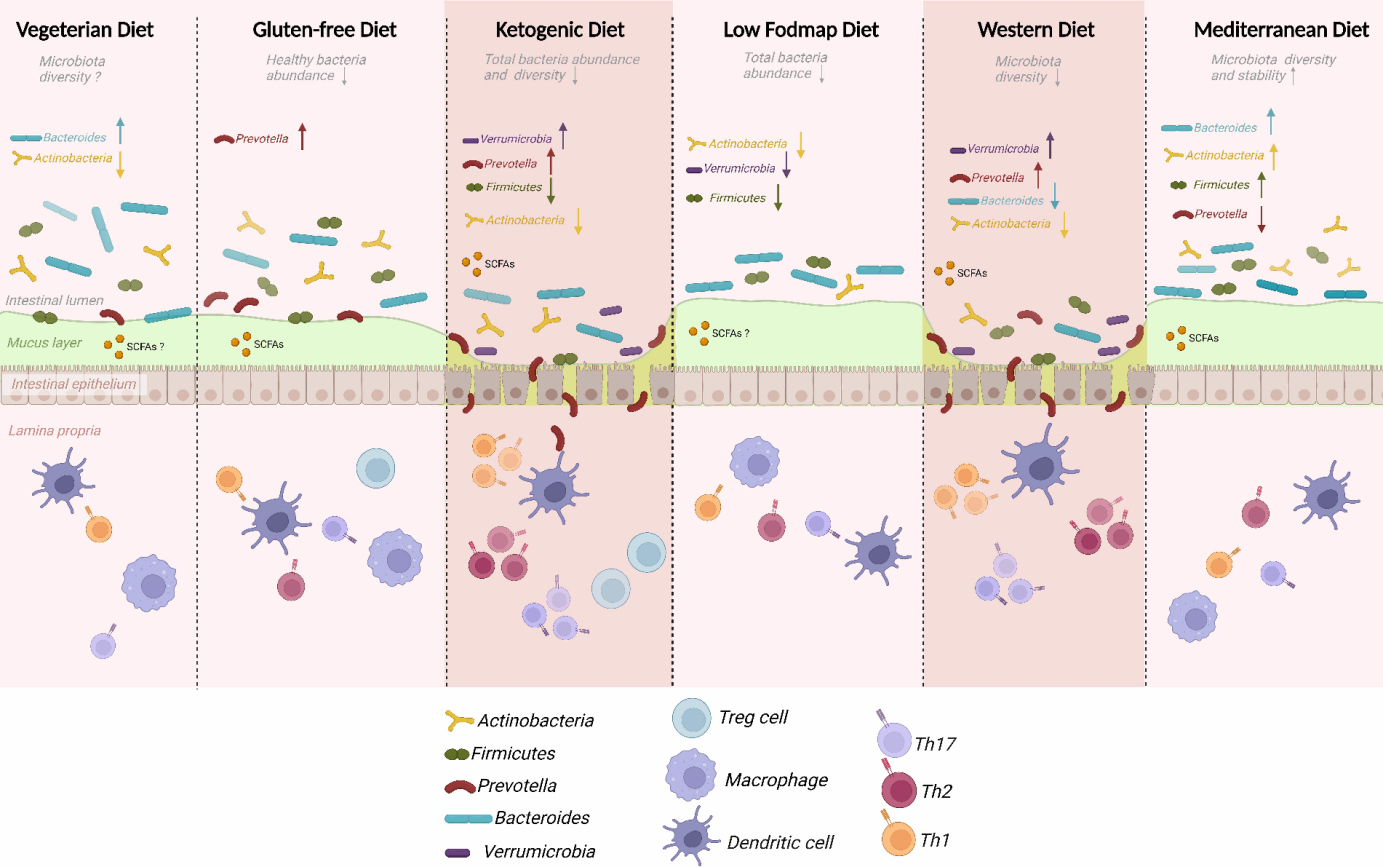
Dietary impact on gut microbiota composition.

The intricate relationship between diet and gut microbiota has emerged as a pivotal determinant in the multifaceted landscape of human health (Figure 1). Both our digestive processes and susceptibility to gastrointestinal disorders are profoundly influenced by the dynamic interplay between dietary components and the microbial inhabitants of the gastrointestinal tract (14).

### Food and bacteria as a complicated Tango

The composition of our gut microbiota is directly influenced by the nutrients we consume. The gut microbiota can respond in various ways to different components of the diet, including carbohydrates, proteins, lipids, fibers, and specific bioactive chemicals. Complex carbohydrates, such as dietary fiber, serve as a crucial source of fuel for certain beneficial bacteria. The fermentation of fiber by these bacteria produces short-chain fatty acids (SCFAs)—which are organic acids produced by gut bacteria during the fermentation of dietary fiber (Table 1), which possess anti-inflammatory properties and contribute to gut health (15, 16). Proteins from the diet can impact gut microbial diversity, with high-protein diets potentially encouraging the growth of bacteria that utilize amino acids, leading to the generation of harmful compounds. The types and quantities of fats in one’s diet significantly affect the composition of the gut microbiome, potentially influencing bacterial imbalances linked to obesity and metabolic diseases (17). Foods rich in fiber and prebiotic ingredients sustain beneficial bacteria and foster a healthy microbiome, playing a crucial role in preventing and treating digestive disorders (18). Polyphenols and phytochemicals, plant-based molecules with antioxidant and anti-inflammatory characteristics, can positively influence the gut flora (19).

**Table 1:** Metabolites from food and their associations with gut microbial communities: additional review points.

Metabolite	Food Source	Microbial association
Short-chain fatty acids (SCFAs)	Fiber-rich foods such as fruits, vegetables, and whole grains	Produced by gut microbiota through fermentation of dietary fiber
Polyphenols	Fruits (e.g., berries), vegetables, tea, red wine	Metabolized by gut bacteria into bioactive compounds with antioxidant and anti-inflammatory properties
Tryptophan	Protein-rich foods such as turkey, chicken, eggs, nuts, and seeds	Metabolized by gut bacteria into serotonin and other neurotransmitters, influencing mood and cognitive function
Sulfur-containing compounds (e.g., hydrogen sulfide)	Cruciferous vegetables (e.g., broccoli, cabbage), garlic, onions	Production by sulfate-reducing bacteria in the gut, implicated in gastrointestinal health and disease

### Consequences for digestive disorders

The intricate dance between nutrition and gut flora has far-reaching effects on gastrointestinal disorders. Changes in gut microbial composition have been associated with conditions such as irritable bowel syndrome (IBS), Crohn’s disease, ulcerative colitis, and gastroesophageal reflux disease (GERD) (20, 21). Dietary habits can either exacerbate or alleviate symptoms, emphasizing the potential for controlling and preventing gastrointestinal diseases through personalized dietary approaches (22).

## Exposing the role of dietary fiber in feeding good bacteria

The significance of dietary fiber in nurturing a robust gut flora and maintaining overall gut health is often underestimated despite its widely recognized positive effects on digestion. This humble substance is more than roughage; it is a vital component of the digestive system’s orchestra, playing a crucial role in: Fiber serves as a powerful prebiotic, feeding beneficial bacteria in the digestive tract, producing SCFAs that reduce inflammation, fortify the gut barrier, and enhance overall gut health (23). Eating a variety of fiber-rich foods promotes a healthy balance of microorganisms in the gut (13). Insoluble fiber from whole grains and vegetables aids in relieving constipation by increasing stool volume, while soluble fiber from oats and lentils helps maintain regular bowel movements.

### The effect of fiber on digestive disorders

Increased consumption of soluble fiber has been reported to improve symptoms in some individuals with IBS (24). Dietary fiber may positively impact inflammatory bowel disease (IBD) by altering the gut microbiome, although the extent varies based on the condition and circumstances. A high-fiber diet is associated with a lower risk of diverticular disease and related issues like diverticulitis. Diets rich in fiber are linked to a decreased risk of colorectal cancer, attributed to regular bowel movements and increased SCFA production (25). Dietary fiber plays a pivotal role in maintaining digestive tract health, significantly influencing the gut microbiome’s composition and function. By incorporating a diverse range of high-fiber foods into our diets, including whole grains, fruits, vegetables, and legumes, we not only improve our health but also provide nourishment for the microbes within us (26). The intricate and mutually beneficial interaction between our food choices and the remarkable biosphere within us is best exemplified by the fiber-microbiome partnership (27).

### Studying the role of probiotic-rich foods and prebiotic fibers

The nutritional conductors of gut health, probiotics and prebiotics, orchestrate a symphony of interactions among the microflora in the digestive tract. The gut microbiome, a dynamic ecosystem, is influenced by various dietary components performing unique yet interconnected roles. Probiotics, beneficial bacteria and live microorganisms, and prebiotics, indigestible dietary fibers, offer distinct benefits: Probiotics restore diversity and balance to the gut microbiota, introducing helpful bacterial strains. Probiotics outcompete pathogenic microbes, reducing the risk of infection and gastrointestinal distress. Some probiotics interact with the immune system beneficially, leading to more even and potentially less inflammatory immune responses. The potential modulation of nutritional and energy metabolism by probiotics (28, 29).

### Feeding the philharmonic with prebiotics

Prebiotics, indigestible dietary fibers providing food for beneficial bacteria, contribute to the synergy with probiotics: Prebiotics selectively target and increase the population of specific beneficial bacteria already present in the gut (30). The fermentation of prebiotics produces SCFAs, benefiting gut health by reducing inflammation, fortifying the gut barrier, and supplying energy for colonic cells. Certain prebiotic fibers help with gut motility by promoting regular bowel movements and relieving constipation. Combining prebiotics and probiotics produces a synergistic impact, enhancing health advantages by simultaneously nourishing beneficial microorganisms (31). The gut microbiota is most at peace and resilient when probiotics and prebiotics work together: Combining the benefits of probiotic-rich meals with prebiotic fibers fosters a more diversified and stable microbiome. Improved intestinal permeability defenses are possible, thanks to prebiotic fermentation contributing to SCFA production. Maintaining stability in the gut microbiota, despite dietary or environmental changes, is facilitated by the synergistic effects of probiotics and prebiotics, protecting against dysbiosis (32). The nutritional symphony benefiting the entire gut flora is produced when probiotics and prebiotics work in harmony. These factors, akin to conductors, lead the microbial orchestra to greater unity, variety, and resistance. Improving gut health by incorporating more probiotic-rich foods and prebiotic fibers highlights the interconnectivity of our food choices and the thriving world of microorganisms within us.

## The unsung hero in gut health

Traditional diets have given way to Western diets, defined by the prevalence of processed and convenience foods. This dietary transformation has significantly impacted the food landscape, prompting increased interest in vegetarian and vegan diets with a focus on unprocessed, natural foods (33). Ongoing research delves into the consequences of these dietary choices on gastrointestinal health and function. The Western diet, characterized by its consumption of processed and sugary foods, initiates a cascade of consequences affecting gut health (34). Notably, a decrease in microbial diversity is observed, potentially contributing to abnormalities in the gut microbiota and an increased susceptibility to gastrointestinal diseases (35). Consuming processed foods high in sugar and unhealthy fats may induce a dysbiotic and inflammatory state in the body, with conditions like IBD and IBS linked to inflammation caused by dysbiosis (36). The gut barrier can be compromised due to a diet rich in sugar and fat, potentially leading to toxin absorption and immune system activation. Conditions associated with altered gut microbiota composition, such as obesity and metabolic syndrome, are exacerbated by Western dietary patterns. In contrast, diets rich in plant-based whole foods, including fruits, vegetables, legumes, and grains, demonstrate several positive effects on gut health (37). Plant-based diets are high in fiber and support a diverse and beneficial microbiome by providing sustenance to various types of beneficial gut bacteria. Whole plant diets exhibit anti-inflammatory effects, potentially alleviating gastrointestinal inflammation associated with conditions like inflammatory bowel disease. The fiber in plant-based diets contributes to a stronger intestinal barrier, reducing the absorption of toxic chemicals into the bloodstream. Weight management and metabolic health are positively influenced by plant-based diets, potentially reducing the risk of obesity-related gastrointestinal diseases. While the disparities between Western and plant-based diets in their impact on gut health are evident, moderation remains crucial (38). A nuanced, plant-based diet that incorporates minimally processed foods can be more effective than a strictly binary approach. Our digestive tract’s state and overall health are intricately linked to the foods we consume. The conflict between Western diets, high in processed foods, and plant-based diets, rich in whole, natural foods, underscores the pivotal role of diet in shaping our gut microbiota and overall health. Plant-based diets, coupled with mindful consumption of processed foods, set the stage for a robust gut microbiota, reinforced gut barrier, and a harmonious connection between our dietary choices and the complex ecosystem within us (39).

## Gut microbiome’s impact on gastrointestinal health

The intricate balance of our intestines is profoundly affected by the gut microbiome, a teeming ecology of microorganisms that inhabits our digestive tract. Microbial changes, disrupting the equilibrium of this microbial population, have been linked to several gastrointestinal problems. The IBS, IBD, and gastroesophageal reflux disease (GERD) are all illnesses that may be exacerbated by these changes: The recent study recruited 100 participants with diverse dietary habits and gut microbiome profiles. Participants were randomly assigned to either a personalized dietary intervention group or a control group following a standard dietary recommendation. The personalized intervention group received individualized dietary plans based on their gut microbiome composition, determined through comprehensive metagenomic analysis. The dietary plans were tailored to optimize the growth of beneficial microbial species and reduce the abundance of potentially harmful microbes. After a 12-week intervention period, fecal samples were collected for microbiome analysis, and participants underwent clinical assessments to evaluate changes in gut health markers. The results demonstrated significant improvements in gut microbiome diversity, composition, and metabolic function in the personalized nutrition group compared to the control group. Moreover, participants in the personalized intervention group reported reduced gastrointestinal symptoms and improved overall well-being. These findings underscore the potential of precision nutrition approaches in promoting gut microbiome health and individualized dietary recommendations for optimal health outcomes (40).

### Microbial imbalances

Catalysts for gastrointestinal disorders. Disturbances in the gut microbiota have been linked to IBS, a condition characterized by stomach pain, bloating, and altered bowel habits (41). IBD is characterized by persistent inflammation of the gastrointestinal system and includes Crohn’s disease and ulcerative colitis (41). The microbial imbalance and decreased diversity known as dysbiosis are hallmarks of inflammatory bowel disease. The immune system’s reaction to a dysbiotic pattern could worsen inflammation and hasten the development of disease. Acid reflux and heartburn are symptoms of GERD, which can be affected by changes in the microbiome. The synthesis of metabolites that have an impact on oesophagal health may be affected by bacterial imbalances in the gut (38). In addition, the lower oesophagal sphincter’s function can be affected by these changes, heightening reflux symptoms.

The link between microbial changes and GI problems is mediated in several ways, symptoms and progression of gastrointestinal diseases can be influenced by inflammation and immunological responses, both of which can be triggered by dysbiosis. Changes in gut microbiota composition can weaken the intestinal barrier, enabling potentially dangerous chemicals to enter the body and set off an immunological response. Changes in microbes can affect the production of metabolites such as SCFAs, which affect inflammation and gastrointestinal health. The creation of neurotransmitters may have consequences for disorders like irritable bowel syndrome, and gut microorganisms play a role in this process (42). Managing microbial changes for gastrointestinal well-being, probiotics and prebiotics are used to increase the growth of beneficial bacteria, restore microbial balance, and reduce symptoms. Low-FODMAP diets for IBS is one example of a dietary intervention that has shown potential in the management of IBS symptoms by targeting certain microbial imbalances (43). Tailoring therapy to specific microbial imbalances may be possible with personalized interventions based on an individual’s gut microbiome profile. The function of the gut microbiome in gastrointestinal illnesses is becoming clearer as our understanding of this complex ecosystem grows. Conditions like bowel disease, and gastroesophageal reflux disease have been linked to changes in the microbiome. The substantial connection between our gut microbiota and gastrointestinal well-being is being uncovered by academics and healthcare practitioners as they gain a better knowledge of these dynamics.

## Decoding the brain-gut axis for holistic health

**Figure 2:**
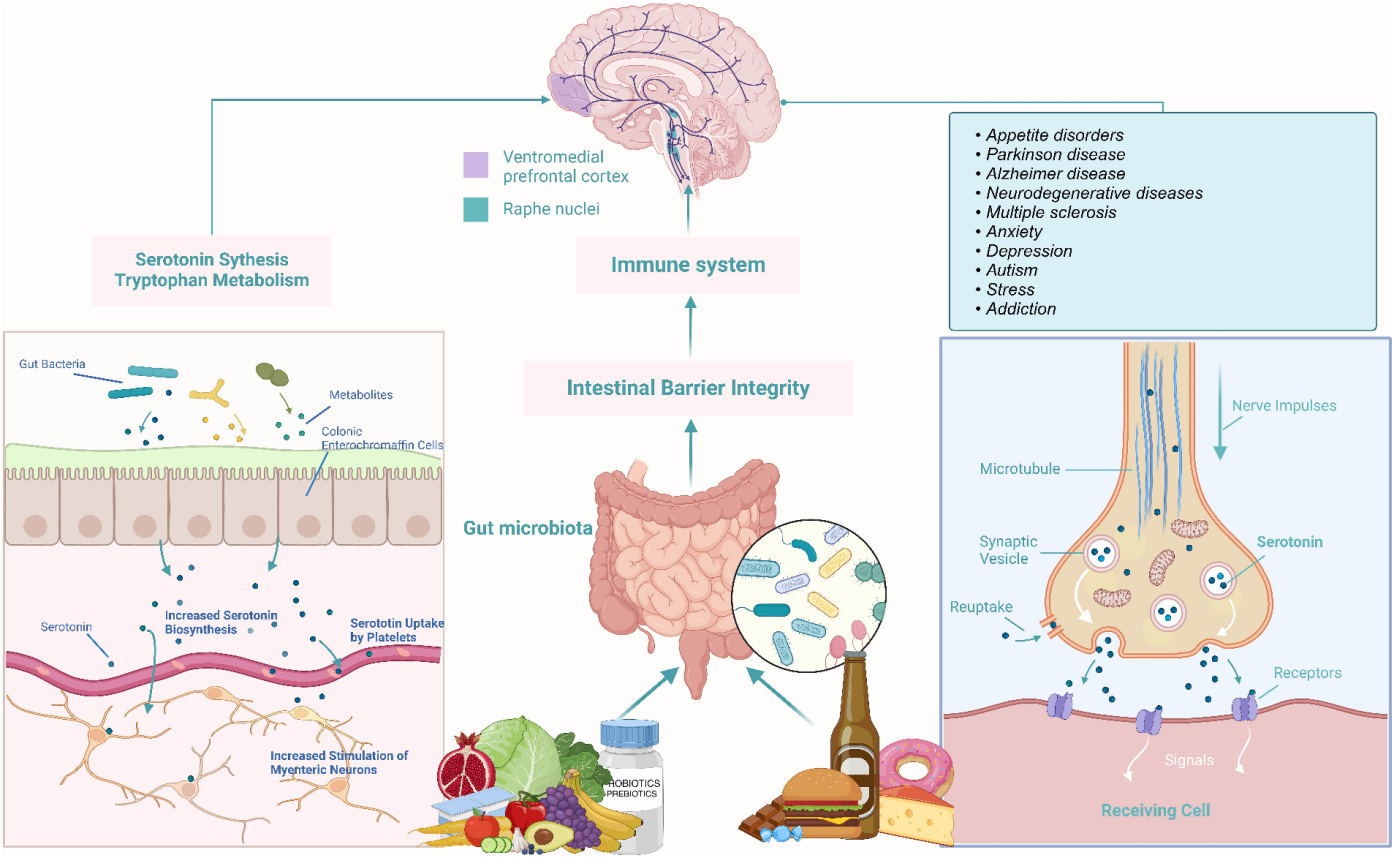
Brain-gut axis: impact of diet on mental health.

The Brain-Gut Axis researches the intricate connection between the gut and the brain, often referred to as the “second brain” (44). This relationship unveils how our food choices impact both physical and mental health. Nutrition emerges as a pivotal player in shaping gut flora, influencing conditions like depression and anxiety. The microbiome in the intestines communicates with the central nervous system, producing metabolites, neurotransmitters, and immunological chemicals. Notably, certain stomach bacteria generate vital neurotransmitters such as serotonin and dopamine (45). A healthy gut flora, nurtured by a diet rich in fiber, prebiotics, and probiotics, contributes to the production of mood-regulating neurotransmitters (Figure 2) (46). Conversely, diets high in processed foods and sugars can induce inflammation, affecting both the gut and the brain and potentially leading to mood disorders (38, 47). The intricate link between the gut and the brain underscores the profound impact of dietary choices on physical, mental, and emotional well-being, emphasizing the importance of embracing nutrient-dense diets and supporting a diverse gut flora for overall health (46).

## Gut microbiome’s role in autoimmune diseases

Autoimmune diseases often manifest in the gastrointestinal tract, with emerging evidence suggesting the crucial role of gut microbiome imbalances in their development (48). Celiac disease, characterized by gluten-triggered immune fibers reactions, showcases the interplay between genetics, the gut microbiome, and disease onset (49). Dysbiosis, or imbalances in the gut microbiome, can trigger immune dysregulation, creating a pro-inflammatory environment that intensifies the immune system’s response to gluten. The consequential changes in microbial metabolite production and damage to the intestinal barrier can lead to conditions like a “leaky gut,” where microbial components pass through, triggering immune responses against the host’s tissues (50, 51). Altered microbial composition, reduced diversity, and abnormalities in bacterial groups are common in the gut microbiota of individuals with celiac disease (49). Recognizing the potential therapeutic role of gut microbiota modulation, interventions like probiotic supplements and dietary changes are explored. Tailoring treatments to individual microbial imbalances offers a promising avenue for managing autoimmune diseases, paving the way for innovative research and potential breakthroughs in understanding and treating these conditions.

## Strategies for reconciling gut microbes through diet

Strategies for reconciling gut microbes through diet play a pivotal role in maintaining digestive tract health. The Low-FODMAP diet, proven effective in managing symptoms of conditions like IBS, targets fermentable carbohydrates to alleviate gastrointestinal issues. Introducing probiotics, beneficial microorganisms, to the diet modulates gut microbiota composition, enhances microbial diversity, and influences immune responses and gut barrier function (26, 40). Prebiotics, found in foods like garlic and bananas, provide indigestible fibers that stimulate the growth of beneficial microorganisms, creating anti-inflammatory short-chain fatty acids (52). The Mediterranean diet, rich in fiber and polyphenol-rich foods, showcases potential benefits against gastrointestinal disorders by enhancing gut flora diversity and balance (53). These dietary interventions offer pathways to microbial reconciliation and improved gut health, underlining the significant role of food in shaping the complex ecosystem of the gut microbiota. As research progresses, these approaches may revolutionize the treatment of gastrointestinal distress, providing personalized and effective strategies for individuals based on their unique microbial composition (54).

## Tailoring diets to microbial masterpieces

**Figure 3:**
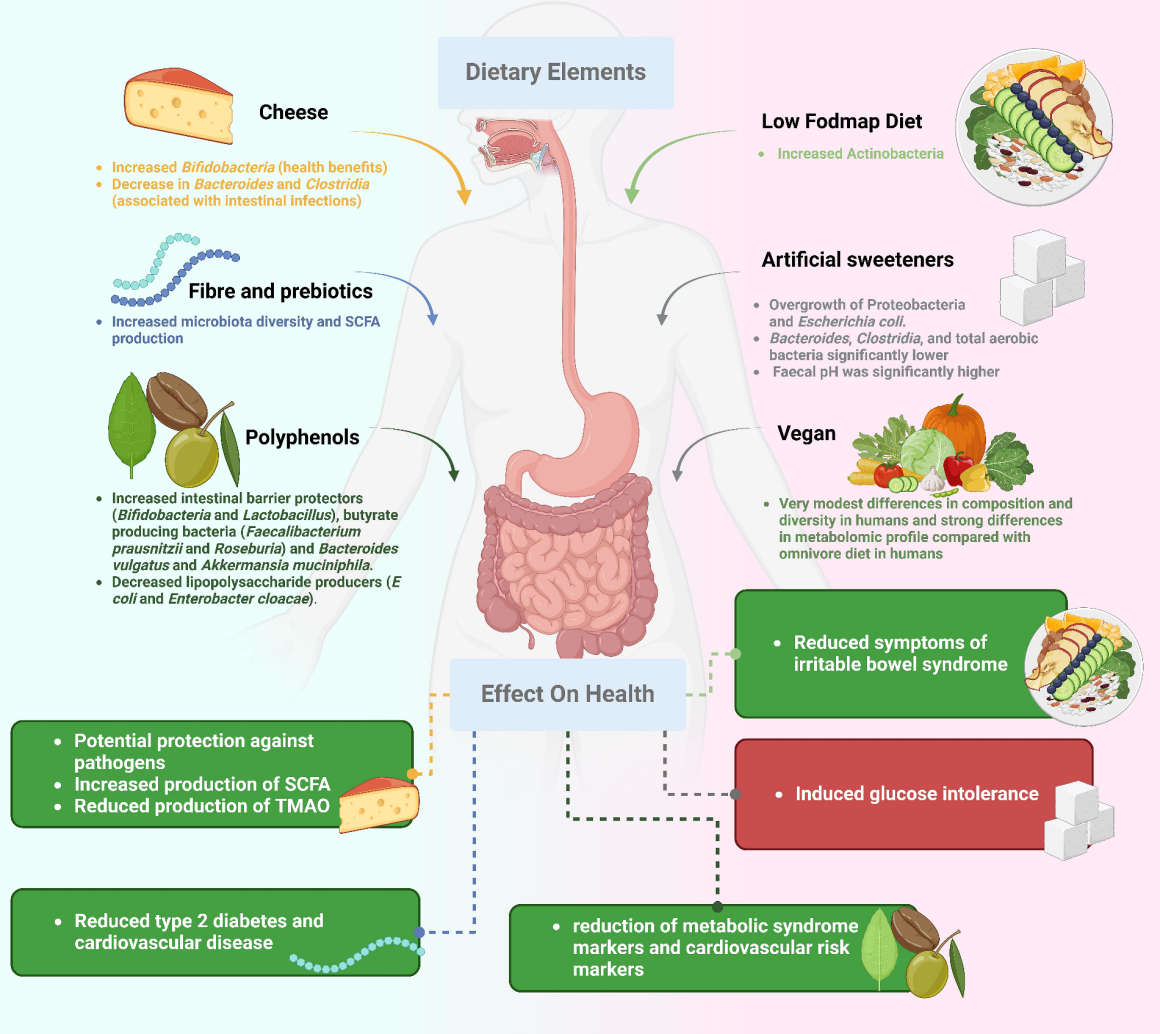
Precision nutrition in gastrointestinal health.

Precision nutrition is a groundbreaking scientific discipline reshaping our approach to health and wellness (Figure 3). By tailoring dietary recommendations to an individual’s unique gut microbiota composition, this emerging field holds the potential to revolutionize the management of gut health and gastrointestinal diseases (55). The interplay of genetics, lifestyle, and gut flora significantly influences how a person responds to food, making personalized nutrition a comprehensive approach. The gut microbiota’s impact extends beyond digestion, affecting various physiological and psychological functions. Profiling the microbiome through modern techniques like metagenomics allows researchers to understand its composition, abundance, and potential health implications. Armed with this microbiome information, healthcare providers can craft personalized nutritional advice, highlighting foods that support good bacteria or those that may disrupt balance (56). Precision nutrition emerges as a powerful tool in modifying the course of gastrointestinal disorders. For individuals with bowel syndrome, tailored dietary strategies based on their unique gut microbiota composition can enhance the effectiveness of interventions like the low-FODMAP diet (57). Similarly, personalized nutrition holds promise in managing IBD by addressing microbial imbalances and identifying specific dietary triggers, leading to reduced inflammation and improved symptom relief. Individuals with food sensitivities can also benefit from precision nutrition by identifying foods that impact their gut microbiota negatively (58). However, despite its potential, precision nutrition faces challenges such as the complexity of microbiome investigation and the need for extensive data interpretation. Direct correlations between the microbiome and health consequences are still under exploration, necessitating further research and development. The shift to precision nutrition for gastrointestinal health represents a departure from conventional approaches, allowing doctors to offer more tailored dietary advice based on an individual’s unique microbiota. This approach exemplifies the synergy between advanced science and personalized care, holding the potential to revolutionize our understanding, treatment, and prevention of gastrointestinal disorders.

## Clinical indications and therapeutic crescendos

Recent groundbreaking studies highlight the pivotal role of gut microbiota in shaping the future of gastrointestinal health. This expanding knowledge has paved the way for novel therapeutic approaches leveraging the ability to alter the gut microbiome through dietary changes and microbial therapies. Specific developments in the field include:

### Exact probiotics

Moving away from blanket approaches, recent probiotic developments focus on tailoring formulations to address unique microbial imbalances associated with individual illnesses. Probiotics designed to produce specific metabolites or regulate immune responses show promise in treating conditions (59).

### Fecal microbiota transplantation

FMT involves transferring feces from a healthy donor to an individual with dysbiotic gut flora. Preliminary results suggest FMT could restore balance in patients with different clinical conditions prompting further investigation into its viability as a treatment (60).

### Microbial metabolites

The fermentation products of gut bacteria, known as microbial metabolites, have diverse physiological consequences. Short-chain fatty acids (SCFAs), such as butyrate, are being studied for their anti-inflammatory properties (7).

### Nutritional software for individual needs

Technological progress is facilitating the implementation of individualized diet plans, contributing to the precision of dietary interventions (61).

### Artificial microorganisms

Scientists are exploring the therapeutic engineering of microbial ecosystems, potentially leading to novel therapies using “designer microbiomes” engineered for specific tasks. Clostridium Cluster XIVa is under investigation for its potential to reduce inflammation and improve gastrointestinal health (62).

### Neuro-gut interventions

Understanding the gut-brain axis opens avenues for new treatments for neurological disorders. Microbiome-targeted interventions, such as modulating neurotransmitters and stimulating the vagus nerve, show promise in addressing conditions like epilepsy and depression. The dynamic world of the gut microbiome’s importance to the future of gastrointestinal health is undeniable. Advances in scientific understanding empower us to modify, engineer, and harness its healing potential. The ongoing journey holds promise for groundbreaking therapies that not only treat symptoms but also address microbial imbalances at the root of many gastrointestinal disorders (44, 45). We stand on the brink of a new healthcare era where the microbial inhabitants of our gut become active partners in achieving optimal gut health and overall well-being. This microbial revolution, driven by precision, innovation, and interdisciplinary collaboration, signifies a transformative shift in healthcare toward personalized and effective treatments for gastrointestinal disorders.

## Conclusion

The exploration of the intricate landscape of the human microbiome has revealed its profound impact on overall health. From the intricate interplay of nutrients shaping microbial diversity to the promising avenues of precision nutrition, this review underscores the evolving understanding of the gut’s pivotal role in human well-being. As we witness the symphony of the gut microbiome, strategies for harmonizing microbial communities through dietary interventions offer tangible solutions for managing gastrointestinal health. Furthermore, the review highlights pioneering clinical approaches, including exact probiotics, fecal microbiota transplantation, and neuro-gut interventions, signaling a transformative shift in healthcare. These advancements not only represent scientific progress but also pave the way for personalized and effective treatments for gastrointestinal disorders. In this symbiotic dance between science and care, the microbial inhabitants of our gut emerge as active collaborators, guiding us toward optimal health. As we stand at the threshold of this new era, the review underscores the importance of precision, innovation, and interdisciplinary collaboration in shaping the future of gastrointestinal health and inspiring avenues for future research and clinical applications.

## Author contributions

ZS: Data curation, Formal analysis, Writing – original draft, Writing – review & editing. LP: Methodology, Project administration, Software, Writing – original draft, Writing – review & editing. SP: Conceptualization, Data curation, Investigation, Project administration, Visualization, Writing – original draft, Writing – review & editing.
